# Understanding dental caries as a non-communicable and behavioral disease: Management implications

**DOI:** 10.3389/froh.2022.764479

**Published:** 2022-08-24

**Authors:** Rodrigo A. Giacaman, Constanza E. Fernández, Cecilia Muñoz-Sandoval, Soraya León, Natalia García-Manríquez, Constanza Echeverría, Sebastián Valdés, Ramiro J. Castro, Karla Gambetta-Tessini

**Affiliations:** ^1^Cariology Unit, Department of Oral Rehabilitation, Faculty of Dentistry, University of Talca, Talca, Chile; ^2^Gerodontology Research Group, Department of Oral Rehabilitation, Faculty of Dentistry, University of Talca, Talca, Chile; ^3^Interuniversity Center on Healthy Aging, Punta Arenas, Chile; ^4^Centro de Epidemiología y Vigilancia de las Enfermedades Orales, University of Chile and University of Talca, Santiago, Chile

**Keywords:** dental caries, non-communicable chronic disease (NCD), dysbiosis, caries management, sugars, interdisciplinary

## Abstract

New paradigms in caries conceptualization have emerged during the last decades, leading to intense debate and discussion on how to approach the disease, both from a preventive and a therapeutic perspective. Among many new ideas, research discoveries and technologies, one major concept can be highlighted that created a deep frontier between the old and the new paradigm in caries conceptualization; the non-communicable nature of the disease, firmly associated with behaviors and lifestyles. This article synthetizes the conceptual construction of dental caries as a non-communicable disease (NCD) based on the current evidence and discusses the appropriate management of the disease in this context. Dental caries has shifted from being considered transmissible and infectious to an ecological and non-communicable disease. Environmental factors such as frequent sugars intake, disrupt the symbiosis of the dental biofilm leading to a dysbiosis, which favors caries lesion initiation and progression. As an NCD, dental caries shares characteristics with other NCDs such as cardiovascular and chronic respiratory diseases, cancer and diabetes, including long duration and slow progression, not being transmissible from person-to-person, being strongly related to modifiable behavioral risk factors, and affecting preferentially disadvantaged populations with a strong inequality gradient. Given the high prevalence of dental caries, and its consequences on people's health and quality of life, a recognizable conceptual view of caries as a NCD is required to target an effective management. Current understanding of dental caries supports prevention through acting on the modifiable risk factors (behaviors) and involves management based on an interdisciplinary approach. Communicating these modern concepts among researchers, clinicians and policymakers is needed to decrease the global high burden of the disease.

## Introduction

Despite the changes in concepts and management strategies during recent years, dental caries is still one of the most prevalent diseases worldwide, with 2.3 billion people having untreated caries lesions in the permanent dentition [1]. One of the most controversial issues is the switch on transmissibility of the disease and the current view as a non-communicable disease (NCD), also known as chronic disease. Until not long ago, dental caries was defined as a communicable infectious disease [2], and understood as an infection of the hard-dental tissues, blaming specific bacteria for its causation [3] (i.e., *Streptococcus mutans* and *Streptococcus sobrinus*). Under that concept, a successful preventive approach aimed to avoid the acquisition and infection with the putative causative microorganisms, that once acquired during the “window of infectivity”, will remain colonizing the mouth. The therapy, once the person was “infected”, encompassed the eradication of the causing bacteria *via* the use of a whole range of antimicrobials or, at the lesion level, through tissue removal, up to sound dentin [4]. However, trying to eradicate specific bacteria from the dental biofilm is a lost battle, as putative pathogens are endogenous to the mouth and will remain as members of the bacterial consortium, regardless of the attempts to eliminate them [5]. Surprisingly, the concept of dental caries as caused by acids from food fermentation and strongly associated with habits, as we conceive it today, was presented more than a century ago, in 1877 [6]. In this article we review the foundations to consider dental caries as an ecological and behavioral NCD.

Dental caries is now understood as an NCD. After the 72nd Session of the World Health Organization's (WHO) World Health Assembly in Geneva, Switzerland in May 2019, the World Dental Federation (FDI) and the International Association for Dental Research (IADR) signed a joint statement for dental caries to be included among the NCDs [7]. According to the WHO, NCDs are those with a long duration, also called chronic diseases. NCDs usually have multiple associated factors that can explain their onset, including genetic, physiological, environmental, and behavioral determinants [8]. Cardiovascular diseases, all types of cancer, chronic respiratory and diabetes are amongst the most common NCDs worldwide. However, accepting the non-communicable character of dental caries is not as extended as other paradigmatic changes in dentistry. This concept implies profound changes in the way the dental profession approaches the disease. Despite the great support for the concept of Minimally Invasive Dentistry worldwide and the fact that most dental schools are switching to conservative approaches for caries lesion management, the idea of caries disease as a NCD and its corresponding management strategies based on that idea, are not equally widespread. Based on evidence, this article attempts to synthetize the conceptual construction of dental caries as a NCD and propose ideas for management, rooted on these conceptual views. Although the categorization of caries as a NCD has been mentioned before in several places, the rationale to support this concept is still weak and partially developed. Hence, we aimed to provide a holistic support for the idea of caries as NCD, using a multidimensional conceptual framework, involving biological, medical and sociocultural components and how they can be integrated into an effective disease management. Furthermore, a more accurate understanding of the nature of the disease will allow the inclusion of caries into the public health policy, along with other NCDs and with the interdisciplinary health team.

## Dental caries is a dysbiosis and not an infection

Technological advancements and scientific evidence allowed the change on the understanding of dental caries, from an infectious to a non-infectious disease. The main foundation for moving away from the infectious pathogenesis is that dental caries does not follow the three canonical Koch's postulates [9]. As presented in Table 1, dental caries hardly qualifies into the category of infectious disease based on multiple facts: [1] the putative pathogen(s) can still be isolated from healthy subjects [2, 10], the mere presence of the putative pathogen(s) does/do not cause the disease. Indeed, pathogenic microorganisms in caries, are also present under healthy conditions, but at lower levels, indicating that they cannot be considered infectious in nature; [3] there is not a single specific bacterium causing the disease. Conversely, dental caries has a polymicrobial nature, induced by commensal bacteria with the potential of causing disease under imbalanced environmental conditions, posed by exposure to frequent and high amounts of sugars. During ecological imbalances, known as dysbiosis, the dental biofilm is stressed by highly acidic conditions, turning commensals into pathobionts, potentially causing the disease [11]. Consequently, the presence of specific bacteria such as *S. mutans*, which was blamed as the pathogen causing dental caries, does not determine the acquisition of the disease [12]. Since oral environmental changes are responsible for the switch from symbiosis to dysbiosis, the type of bacteria in the dental biofilm becomes less important than the overall metabolic activity of the bacterial consortium under sugars-rich and acidic conditions [13]. Indeed, competition within the species of the biofilm is dependent on the conditions imposed by the presence of sugars [14, 15].

**Table 1:** Side-by-side comparison between Koch's postulates [9] and dental caries, that shows why dental caries does not follow the pattern of canonical infectious diseases.

**Koch's postulates** ** (infectious disease)**	**Dental caries** ** (non-communicable disease)**
The **microorganism must be found** in abundance in all organisms suffering from the **disease** but should not be found in healthy organisms.	Putative pathogen microorganisms detected in **disease are also present** under **healthy conditions**.
The **microorganism must be isolated** from a diseased organism and grown in pure culture.	There is not a specific bacterium causing the disease. It is **polymicrobial**, caused by pathobionts (commensal bacteria with the potential of causing disease under dysbiosis).
The cultured **microorganism should cause disease when introduced** into a healthy organism.	The mere **presence of the bacteria does not cause the disease**.
The microorganism must be reisolated from the inoculated, diseased experimental host, and identified as being identical to the original specific causative agent.	There is **not a specific bacterium** causing the disease.

### Dental biofilm: Necessary, but not enough to cause dental caries

The oral cavity is a complex ecological system, strongly colonized by a rich consortium of microorganisms comprising bacteria, viruses, protozoa, and fungi, inhabiting small microbial niches on the teeth, and other regions of the oral cavity [16]. Bacterial communities found in the mouth are extraordinarily complex. These microorganisms encompass what is called the oral microbiome, that include commensals symbiotic and pathogenic microorganisms. Oral microbiota are normal residents of the mouth that can become pathogenic upon environmental disturbances. DNA-based studies of microbial diversity have estimated that the oral biofilm contains between 500 and 700 bacterial species [17, 18]. Furthermore, there is wide variation in the bacterial phenotype and metabolic activity among species of the oral microbiome. As discussed above, changes in the biofilm are due to the oral and biofilm microenvironment circumstances. Thus, the sole presence of a biofilm, ubiquitous to the tooth surface, will not determine the disease.

### Role of sugars in driving the dysbiosis

Although defined under the ecological plaque hypothesis almost two decades ago [19], the role of diet in caries has been highlighted only in recent years [20]. Diet drives and determines the onset of caries disease. Sugars-rich diets induce the formation of cariogenic biofilms, which increase the risk of developing lesions. Under the presence of sucrose and other fermentable carbohydrates (mainly simple (free) sugars, i.e., mono and disaccharides), the ecological balance or symbiosis (health) moves toward an ecological dysbiosis (disease) [21]. Thus, sugars should be considered the driver of caries disease [22–24]. Cariogenic and active biofilms accumulate great amounts of acids that are released into the dental biofilm fluid, breaking the mineral equilibrium in the enamel or dentin. Under these acidic conditions, the homeostasis of the dental biofilm is lost, shifting the net mineral gain to a net mineral loss [25]. An inappropriate intake and balance of nutrients triggers that typically cariogenic species [S*treptococcus mutans (Sm), Lactobacillus, Scardovia wiggsiae and Actinomyces* spp.] predominate over those mostly associated with health (*S. sanguinis, S. oralis*, and *S. mitis*) [3, 5, 14, 25–29]. On the other hand, some bacteria can raise the pH by producing ammonia from urea and arginine, providing a mechanism for balancing acid production to maintain homeostasis [15, 30].

## Shared characteristics of dental caries and other NCDs

Unlike communicable infectious diseases that have a rapid onset and progression, NCDs are also called chronic diseases, due to their long duration and generally slow progression. No consensus has been reached about the time needed to consider a disease as chronic, but general agreement indicates that any illness that lasts longer than 6 months can be considered chronic. Hence, and since dental caries lesions slowly progress over the years, caries may be considered as a chronic disease. It has been reported that a newly formed caries lesion may compromise the entire enamel, in a range of 6–6.3 years [31, 32].

Most common NCDs include cardiovascular diseases, cancer, respiratory diseases, and diabetes, and are responsible for more than 60% of deaths, constituting the main cause of mortality in the world with more than 40 million deaths per year [33]. NCDs contribute about 80% of the total burden of disease, resulting in exorbitant economic costs and a major public health issue. Although most of the NCDs do not share the same etiological factors, they have similar health risk behaviors, such as high sugars consumption, alcohol drinking, tobacco use, malnutrition and low physical activity or sedentarism. All these factors are deeply related to psychological, social, cultural, and economic factors [34]. Since the main etiological factor in dental caries is sugars consumption, poor dietary habits, and deficient biofilm control, it is plausible to argue that the disease shares common risk factors and/or etiology with other systemic NCDs, for example diabetes and obesity (Table 2). Indeed, a recent WHO report on oral health states the need to address “the common risk factors of oral diseases and other NCDs through an integrated approach, focusing on key risks, such as tobacco and harmful alcohol use, unhealthy diets and poor hygiene” [35].

**Table 2:** Parallel comparison between general characteristics of common non-communicable diseases (NCDs) and dental caries.

**General characteristics of NCDs**	**Dental caries**
Chronic diseases are diseases of long duration, >6 months.	Caries is long-lasting and can be present throughout the life course
Slow progression	Caries lesions may take years to progress in extension from one stage to the other (i.e., enamel to dentin)
Progressive deterioration	At the tooth level, caries lesion progression leads to a gradual tissue loss and impaired oral function
Contribution to high morbidity and burden of disease	Untreated caries is one of the most common conditions of humans, affecting at least 35% of the population.
High economic burden for the patient, their family, and the Social Security System	Restorative management of dental caries require high economic expenses, with restricted access and usually difficult to afford for the vulnerable population.

Another trait of most NCDs is that they are preventable. Prevention of NCDs typically imply a reduction of associated risk factors [36]. For example, preventive strategies for type 2 diabetes and cardiovascular disease involve behavioral change toward a healthy diet and lifestyles [37]. Caries management should follow the same scheme and emphasize prevention by intervening behavioral factors over the restorative treatment of the already created caries lesions. Acting on lesions is too late as a preventive strategy. Regardless of the benefits of using minimally invasive therapeutic approaches to tackle caries lesions, a successful preventive program should aim at correcting health behaviors to avoid the biofilm dysbiosis, before the onset of any detectable clinical sign of the disease.

## Systemic health and dental caries

Recent and ongoing investigations point toward an influence of oral health on systemic conditions and vice versa, with an accepted bi-directional relationship [38]. Caries disease is linked to morbidity and death through tooth loss. Dental caries is the main reason for tooth loss, so adding tooth loss to untreated dental caries increases the burden of oral disease [39] and the impact of caries on systemic health. Dental caries may affect people from early life and usually persists over the years until death. Recent studies have shown that tooth loss, irrespective of the cause, is associated with the incidence of other NCDs, such as myocardial infarction, heart failure, ischemic stroke and death from all causes, becoming a good predictor of cardiovascular outcomes [40]. The association of tooth loss seems stronger with myocardial infarction followed by heart failure stroke and overall mortality. In fact, having ≥5 missing teeth substantially increased the risk for cardiovascular outcomes, and even a small number of missing teeth [1–4] was associated with an increased risk for myocardial infarction, stroke, and death [40].

Though the link between tooth loss, derived from caries, and cardiovascular disease is consistent, causality is not completely elucidated, but it could be attributed to chronic low-grade inflammation of oral tissues, as inflammation contributes to the pathogenesis of atherosclerosis [40]. Likewise, the underlying mechanism in the association of dental caries and coronary heart disease remains unclear. The penetration of oral bacteria through cavitated caries lesions causes chronic infection and inflammation of the dental pulp without treatment and the periapical tissues [41]. Severe dental caries may cause chronic inflammatory response in the endothelial coronary cells through bacterial invasion from decayed teeth [42]. Also, severe dental caries can induce atherosclerosis and exacerbate cardiometabolic risk factors that contribute to the onset of coronary heart disease [43]. These biological mechanisms can be responsible for the association between dental caries and coronary heart disease. Yet, better designed studies with more controlled factors are necessary to confirm such association [42].

Caries has also been associated with cognitive impairment and dementia [44]. It is well-known that people with dementia may lose the capacity to maintain an appropriate oral hygiene, resulting in more dental biofilm stagnation, thus, increasing their risk of developing periodontal disease and dental caries. The inverse directionality of the association, that is, dental caries and tooth loss as an explanation for cognitive impairment and/or dementia is less explored and still insubstantial. Further research to test these associations result of interest and should be considered in future research initiatives. If a causative role of dental caries is demonstrated for cardiovascular and/or neurodegenerative disorders, oral health should be more decidedly incorporated within the group of NCDs, with an interdisciplinary approach (see below).

## Comprehensive management of dental caries as a NCD, beyond the tooth

Dental caries is profoundly rooted in health risk or unhealthy lifestyle behaviors, such as high sugars consumption which is also related with low income [45, 46]. A lifestyle is the way people live and is strongly associated with the behavioral and motivational spheres of human beings [36, 47]. Considering known risk factors, such as low socio-economic [45, 46], high sugars consumption and other unhealthy lifestyles [48, 49], these risk factors are key pieces for preventing and managing NCDs, at a global scale. To achieve a healthy life, several changes in habits have been recommended [50]. While overall healthy behaviors should be applicable too, in dental caries a poor nutrition with excess of sugars and an inadequate biofilm control are the most relevant behavioral and modifiable factors [51, 52]. In fact, compelling evidence has demonstrated that poor health behaviors are correlated with a higher prevalence of dental caries [53–56]. As previously described, there are substantial reasons to worry about controlling dental caries disease. As dental caries is the result of interactions between biological, behavioral, sociocultural and contextual factors [57], approaches to manage dental caries should be at both the individual, and the population level.

### Management at the individual level throughout the life course

The canonical approach for managing caries at the individual level considering only the oral factors and restorative treatments have failed to show results at a large scale, especially in vulnerable populations. New strategies, therefore, must be taken to incorporate behavioral factors and lifestyles into any preventive dental care program [58]. Understanding dental caries as a NCD expands the scope to prevent and to treat the disease, putting diet and healthy behaviors in the center of its management. For example, preventive strategies for type 2 diabetes and cardiovascular disease comprise a behavioral change toward a healthy diet and lifestyles [37]. Furthermore, it has been reported that more than one health risk behavior acting together on an individual, impact health much more significantly than single poor habits [59]. Thus, managing dental caries as a NCD strongly invites to using different strategies to those currently in use, to be effective in controlling the disease and its consequences. Overall healthy behaviors should result in oral health benefits. Thus, drinking tap water instead of sugar-sweetened beverages (SSB) or sugary beverages will impact not only on lower caries incidence, but also in general health outcomes. In fact, frequent intake of SSBs has been vastly associated with weight gain and obesity [60], and also with metabolic syndrome and type 2 diabetes [61].

It is important to highlight and convey the message that a restorative approach to treat caries lesions only limits the damage already present, but it does not reach the disease that caused them. Caries management should emphasize prevention by intervening behavioral factors. Instead of focusing preventive efforts in changing lifestyles with sugars intake restriction and counseling on healthy lifestyles, the dental profession has prioritized preventive programs on strategies aimed at interfering with the demineralization and remineralization process, contributing to reduced net mineral loss, at the tooth level (mostly with fluorides). This approach falls more into an infectious conceptual view of the disease rather than on a NCD nature, as discussed here. For example, the “trendy” use of silver diamine fluoride will not control caries disease in its genesis, but the lesions in their progression [62]. Hence, putting too much attention on measures to cope with the caries lesions will diverge the focus on the origin of the problem and may lead to erratic public policy strategies.

Like in type-2 diabetes where poor disease control will lead to worsened signs and symptoms over time, the lack of control and monitoring over time will lead to lesion progression and loss of the “biological asset” [63]. In fact, a restoration cycle or “spiral of death” [64] has been described to portrait the natural course of a small restoration performed during early years, that after a repetitive cycle of restoration and re-restoration, ends up in tooth loss at more advanced ages. One of the goals of the dental profession should be, in fact, to preserve a functional dentition throughout the life course [65].

Based on the current understanding of the disease, mechanically controlling the biofilm by toothbrushing should not be the sole milestone for caries management. It is true that infrequent toothbrushing has been related with more severe carious lesions than those with better hygiene habits [66], but dietary patterns and nutrition are progressively receiving more attention by researchers and policymakers in dental matters. Yet, toothbrushing persists as the most relevant component in any preventive dental care program and sugars restriction typically is approached by just saying: “do not eat sugar”. The WHO recommends to reduce the consumption of simple sugars to <5% of the total daily energy intake, based on the best evidence [67]. For children under the age of two, the recommendation is to avoid adding sugars to food or drinks. Furthermore, a balanced diet containing other macromolecules (e.g., proteins) can be protective for dental caries [68], and it should be promoted. Additionally, fiber-rich foods might act as caries-protective factors *via* an ecological therapy that modifies the dynamics of the dental biofilm.

Another factor related with dental caries and behavioral factors is the provision of dental care. Regular dental checkups have shown as important to maintain good oral health [69]. Thus, caries management at the individual level, should include dietary counseling with sugars intake restriction, biofilm control and periodic checkups according to risk to promote oral and systemic health. Despite the latter are desirable goals in any caries preventive program, dental illiteracy may be an important barrier [70]. Talking to a patient “teaching” them how to prevent the onset of caries is a common practice. Whether this is effective in controlling causative factor, results less clear. Dentists should use tools that had shown effectiveness in changing deleterious habits and reducing the incidence of caries lesions. In that context, Motivational Interviewing to generate behavioral change appears necessary to manage the disease. This technique has been used in caries preventive programs with better results than traditional education techniques [71]. The extent and effectivity of the Motivational Interviewing in preventing caries has been recently systematically reviewed [72], showing that in high caries populations, this technique is helpful in increasing knowledge and changing oral health behaviors, resulting in lower early childhood caries experience, and also improving oral health behaviors and caries in adolescents [73].

Health risk behaviors in caries may not be transmissible as an infection with exogenous pathogens, but deleterious health-related behaviors can be passed on from one individual to another or acquired from social and cultural beliefs [74]. Thus, although there is evidence to support dental caries as a chronic or non-communicable condition, it is necessary to highlight and teach patients that disease-inducing habits are transmissible within the family and social environment, and they should be part of any caries preventive program. Dentists will not be effective in controlling dental caries if only tooth interventions are performed, omitting the non-communicable nature of the disease, strongly associated with lifestyles and behaviors.

The long-lasting course of all NCDs makes it necessary to monitor affected people throughout the life course. Against current preventive practices focused on children, adults and older persons must be also included, as caries is a lifetime chronic disease that continues to affect the person throughout life [75]. The idea of monitoring NCDs from an early age, for example in heart conditions [76], is a common objective applicable to all NCDs, including dental caries disease. Thus, the focus in promoting health and controlling, instead of curing, the disease should be widely acknowledged. Furthermore, teledentistry using new telematic tools could be adopted more frequently [75], as they have proven effective in preventing and promoting oral health [77], at a lower cost and facilitating access to health education and counseling to people living in remote areas or with several other barriers. The latter is especially true during pandemic times, as restricted physical mobility needs to be ensured. However, to be effective in caries control, individual measures must be combined with public policy and oral health care programs at the community level.

### Managing dental caries at the community level

Since habits can be considered “communicable”, focusing on early interventions during pregnancy and on small children, with an emphasis on diet and nutrition must be a priority in elaborating public policies in health. To effectively manage dental caries, public policies are extremely important to allow populations to have better life conditions and to maintain healthy lifestyles. Modern societies, however, promote a whole range of behaviors in the population. For example, marketing on food and clothing reaches large populations influencing their decision and lifestyle behaviors. Moreover, lower socioeconomic status typically means restricted access to healthy lifestyle choices, including food.

Regarding dental caries, public policy and community measures for caries prevention and control have focused on fluoridation programs and dental sealant applications [78]. Effective national programs, at the community level, should tackle the causative (sugars and the dental biofilm), instead of acting only on modulating factors. Hence, dental caries must be incorporated among the group of NCDs that have sugars intake as the common risk factor. Examples of good practices and positive public policies include, spreading better and updated information (oral health literacy), higher access to healthy foods, and “banning” unhealthy options by processed food labeling [79] or sugar taxation [80]. Indeed, Chile implemented a comprehensive program for restricting marketing directed to small children of products high in sugars, sodium and saturated fats, with promising results [81].

Since sugars consumption is the main responsible for dental caries and its consumption is widespread and advertised, it is difficult or almost impossible to ask children and adults to make healthy choices when peer, social and market pressure are pushing in the other direction. The solution for dental caries, as well as for most NCDs does not uniquely reside on individual approaches, but on strong policies that can tackle the social, political, and cultural aspects associated with health risk behaviors, drivers of these diseases. Thus, treating dental caries as a NCD will ultimately result not only in lower caries rates, but will also promote healthy lifestyles that in turn, will reduce the prevalence of other NCDs. Viewing dental caries as a NCD represent an opportunity to increase the effectiveness of caries prevention programs. Hence, combining lifestyle measures focused on sugars restriction—at the individual and community levels—with preventive activities targeted at the tooth level (sealants and fluorides) should result in more meaningful and large-scale outcomes.

## Interdisciplinary approach for caries management

A more comprehensive and holistic approach in caries conceptualization will incorporate the disease among other NCDs, such as metabolic disorders, obesity, and type 2 diabetes, all of them having high sugars consumption as one of the etiological factors. Dietary habits and health risk behaviors become common risk factors for this group of NCDs [82]; hence, reducing sugars consumption is a complex task, as it is deeply rooted in social patterns and lifestyles. The high complexity of all NCDs and particularly dental caries, highlights the need for considering theories and multidimensional models that use interdisciplinary and participative approaches to treat, integrating oral health into the interdisciplinary team work [83]. It is already natural to think of an interdisciplinary teamwork for managing type 2 diabetes, with a whole range of professionals participating at different levels. However, it is also common to observe that health care for people with one or more NCDs is provided by multiple professionals, typically without coordination and communication among them [84].

To effectively manage dental caries, a collaboration between dentists, physicians and other members of the health team is required. Having an interdisciplinary approach to one of the most prevalent oral diseases worldwide appears as a potent strategy. Although dentists are not exempt from rising awareness and implementing dietary assessment and counseling as part of their daily work [85], a collaboration with psychologists, nutritionists and other professionals should be part of the complete pool of strategies to implement healthy food choices more effectively. Furthermore, sugars consumption has been associated with additive behaviors and emotions, with a strong neurobiological support [86]. Hence, managing sugars consumption should encompass other health professions to comprehensively approach a complex and multidimensional problem. It is easy to anticipate that isolated dentists working in a dental office and making efforts to reduce sugars intake will be a cumbersome or almost impossible goal, without engaging the interdisciplinary teamwork. On another edge of the problem, training dentists in dental schools to be more competent in using motivational interviewing tools and working with the health team, rather than focusing only on restorative aspects, should also be part of the multidimensional changes needed to alleviate the burden of caries disease worldwide.

Different studies have addressed the effects of interdisciplinary preventive programs to manage caries, focusing on the pregnant mother or children during early childhood. These programs involve different professionals, encompassing educators, gynecologists, midwives, pediatricians, dentists, nutritionists, social workers, and public health administrators [87–89], with promising results [83, 89–91]. Besides the obvious benefit of decreasing caries prevalence, an interdisciplinary approach for oral health and for caries management also allows a better reach to vulnerable populations, who otherwise have limited access to conventional dental treatment. The old paradigm that oral health is restricted to dentists and considered a separate entity, makes that oral health is usually not considered in a routine medical examination. Moreover, despite the importance of an integrated approach for overall health care, oral health is frequently excluded from the therapeutic management, probably due to the historic separation between medicine and dentistry and to a lack of knowledge on the NCD nature of dental caries. The latter may contribute to worsening systemic conditions. Given the traditional division between dental and medical care, including physical locations, communication among health care professionals must be optimized. The interdisciplinary approach for oral health requires efficient communication and bi-directional knowledge by the health care team. While dentists need to be more knowledgeable about systemic health, the health care team needs to know more about dentistry and how oral conditions can affect systemic health.

## Conclusions

**Figure 1:**
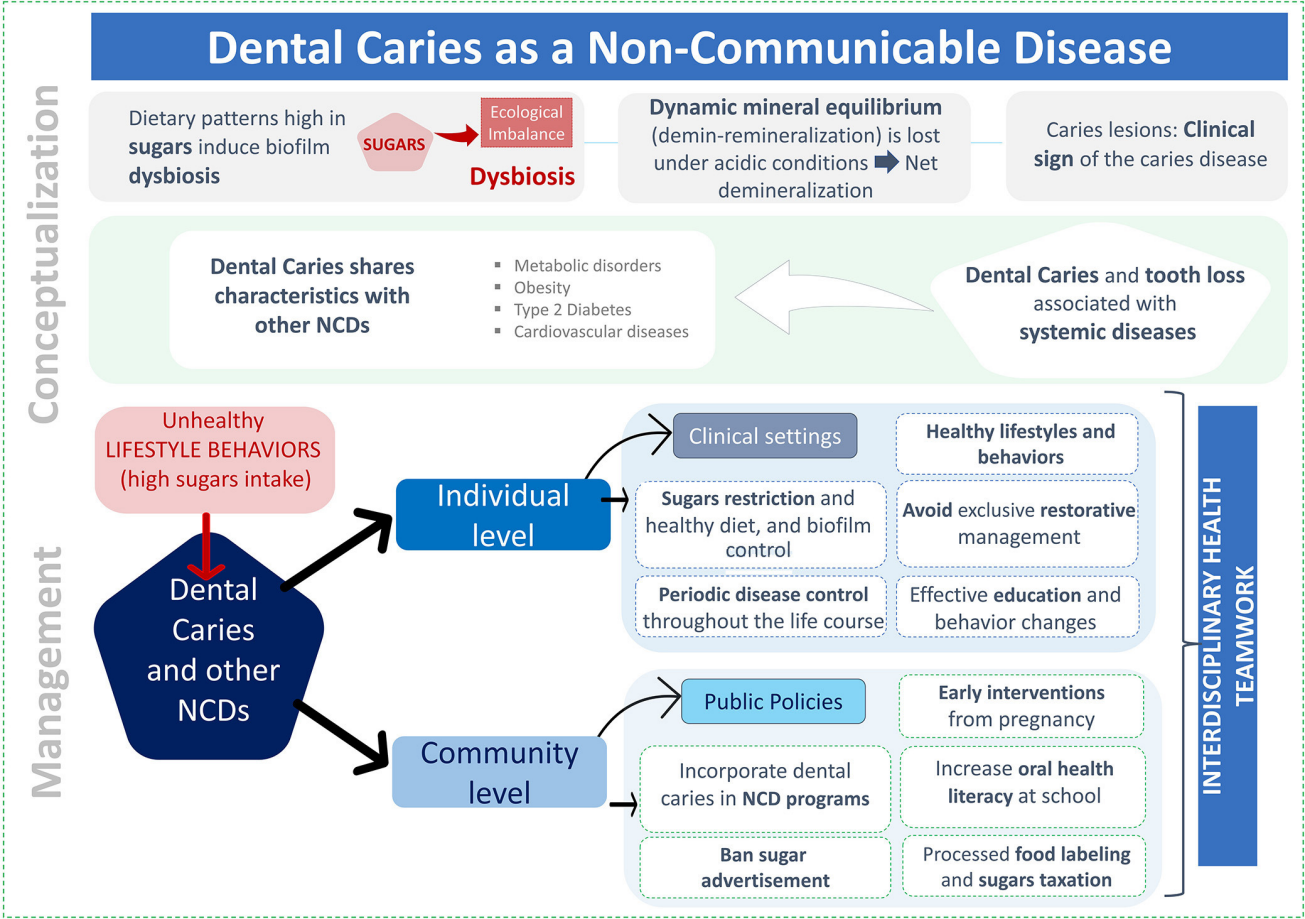
Summary of the conceptual view of dental caries as a non-communicable disease and its management. The upper panel depicts the conceptualization of caries as a non-communicable disease. The bottom panel represents a schematic view of the proposed caries management, at the individual and community levels, both with an interdisciplinary approach.

Dental caries must be considered non-communicable, associated with a dysbiosis of the dental biofilm and caused by free sugars exposure, but strongly linked to deleterious lifestyles and behaviors, mainly related to inappropriate dietary patterns. Modern management of the disease, therefore, should comprise acting at the individual and the community levels, incorporating caries among the public health policy for NCDs (Figure 1).

Although dentists are primarily responsible for caries management, the complex and multidimensional nature of this NCD requires the engagement of the interdisciplinary health teamwork to expand and enhance the health outcomes, beyond oral health. An appropriate inclusion of dentists and oral health in the interdisciplinary health team and in public health policies will allow comprehensively tackling caries and other NCDs, resulting in sustained healthy lifestyles, especially in those related to dietary habits.

Treating caries as a NCD becomes a novel paradigm for the dental profession, that will contribute to remediate the excessively high burden of dental disease, impacting overall health and improving quality of life, especially for those most vulnerable populations worldwide, at all ages.

## Author contributions

RG: conception, design, and draft of the first manuscript. CF: revision of the first manuscript and incorporation of ideas and edition. CM-S, SL, NG-M, CE, SV, RC, and KG-T: contribution of initial ideas and manuscript revision. All authors gave their final approval and agree to be accountable for all aspects of the work.

## Funding

This study was funded by two Chilean Government Grants (ANID): Fondecyt Regular 1210188 to RG and Fondecyt Iniciación 11200431 to SL.

## Conflict of interest

The authors declare that the research was conducted in the absence of any commercial or financial relationships that could be construed as a potential conflict of interest.

## Publisher's note

All claims expressed in this article are solely those of the authors and do not necessarily represent those of their affiliated organizations, or those of the publisher, the editors and the reviewers. Any product that may be evaluated in this article, or claim that may be made by its manufacturer, is not guaranteed or endorsed by the publisher.
